# Techniques for direct experimental evaluation of structure–transport relationships in disordered porous solids

**DOI:** 10.1007/s10450-016-9806-9

**Published:** 2016-08-13

**Authors:** Artjom Nepryahin, Robin S. Fletcher, Elizabeth M. Holt, Sean P. Rigby

**Affiliations:** 1 Present Address: Department of Chemical and Environmental Engineering, University of Nottingham, University Park, Nottingham, NG7 2RD UK; 2 Johnson Matthey, P.O. Box 1, Belasis Avenue, Billingham, Cleveland, TS23 1LB UK

**Keywords:** Mass transport, Bimodal support, Gas sorption kinetics, Mercury porosimetry

## Abstract

Determining structure–transport relationships is critical to optimising the activity and selectivity performance of porous pellets acting as heterogeneous catalysts for diffusion-limited reactions. For amorphous porous systems determining the impact of particular aspects of the void space on mass transport often requires complex characterization and modelling steps to deconvolve the specific influence of the feature in question. These characterization and modelling steps often have limited accuracy and precision. It is the purpose of this work to present a case-study demonstrating the use of a more direct experimental evaluation of the impact of pore network features on mass transport. The case study evaluated the efficacy of the macropores of a bidisperse porous foam structure on improving mass transport over a purely mesoporous system. The method presented involved extending the novel integrated gas sorption and mercury porosimetry method to include uptake kinetics. Results for the new method were compared with those obtained by the alternative NMR cryodiffusometry technique, and found to lead to similar conclusions. It was found that the experimentally-determined degree of influence of the foam macropores was in line with expectations from a simple resistance model for a disconnected macropore network.

## Introduction

Heterogeneous catalysis is a key sector of high technology manufacturing. Maintaining competitiveness of the catalyst industry requires the continued development of ever higher-performance products, delivering improved catalytic activity and chemical selectivity. For products catalysing diffusion-limited reaction systems, increasing the pellet effectiveness factor is key to delivering improved performance. This requires a detailed understanding of the factors in pellet pore structure that limit mass transport, and the parameters in the manufacturing process that produce these particular pore structure features.

Catalyst particles can be formed in a number of different ways, including granulation, extrusion, and pelleting. Further, the product of catalyst pelleting, for example, is governed by a number of different process parameters such as mould-to-particle size ratio, uni-axial versus bi-axial compression, type and amount of lubricant, and compaction pressure. The required post-compaction treatments, such as calcination, also present a choice of parameters, such as calcination atmosphere and temperature. Catalyst pellets often also undergo further treatments, such as in situ reduction, before final end use on the customer’s plant, which also offer more choice amongst preparation conditions, such as reductant type and temperature. Similarly large sets of choices are available for other forming methods. The vast extent of the range of options means that purely empirical methods of process design are likely to be time intensive and expensive for finding optimal conditions and parameters, and thus a more efficient, systematic approach is required. Proper understanding of the impact of manufacturing process parameters is likely to facilitate more optimal choices of these parameters.

In the past understanding (as opposed to just measuring) the impact of the manufacturing process on the pellet performance has involved detailed structural characterization of the pore network produced by a given manufacturing process, and using the inputs from this analysis to construct a computer model of the void space on which to simulate mass transport and reaction processes. This approach requires accurate structural characterization and the ability to build a sufficiently representative computer model to deliver predictive simulations. However, accurate structural characterisation of porous media is limited by complex effects such as advanced condensation in gas adsorption (de Boer 1958; Hitchcock et al. 2014), pore shielding in mercury porosimetry (Androutsopoulos and Mann 1979; Matthews et al. 1995), and advanced melting in thermoporometry (Hitchcock et al. 2011). Development of pore structural models on computer, which are truly statistically representative of real porous solids, is limited by the observation that many materials possess structural heterogeneities over the full range of length-scales from molecular scale (~0.1 nm) to pellet scale (~1 to 10 mm), including such features as pore surface roughness, different network geometry, and macroscopic spatial distribution of pore size and porosity. This heterogeneity often means that the small sub-section of the pellet that can be simulated on computer does not accurately reflect the whole. These limitations necessitate the adoption of a different strategy to understand structure–transport relationships.

This work will thus use newly developed methods that directly reveal the structure–transport relationship of the pore structure. These methods use controlled techniques to selectively block particular portions of the pore structure and determine the transport properties before and after this removal. Complementary computerised x-ray tomography (CXT) allows the visualisation of the spatial distribution of the blocked-out void-space regions for materials with macroscopic heterogeneity (which is typical). This procedure allows the degree of influence, on transport properties, of the removed pore network portion to be determined. The techniques involve use of mercury porosimetry to selectively remove (typically) larger pore sizes via mercury entrapment. The initially empty, and subsequently partially-blocked, void space is probed by gas uptake at temperatures below the pore-blocking fluid freezing point, such as nitrogen at 77 K. In addition, cryodiffusometry (Shiko et al. 2013) can be used to selectively freeze-out different sets of larger pores and study molecular self-diffusivity in the remaining smaller pores. These techniques will be used to evaluate the impact of the various structural features produced by processes involving different sets of manufacturing parameters and, thereby, deduce the relationship between manufacturing route and material effectiveness.

Many catalytic processes have plant operating conditions such that pore diffusion in mesopores is in the Knudsen regime. In this case a low pore size has a severely limiting effect on mass transport rates. However, using larger pore size materials means a lower surface area-to-volume ratio of the void space and thus a decline in catalytic activity due to less active surface area. A common approach (Mann and El-Nafaty 1996; Gheorghiu and Coppens 2004) adopted to try to achieve an optimum balance between these extremes is to use a bimodal (or bidisperse) pore structure, with a pervasive macropore network to provide rapid ‘motorway/freeway’ access to the centre of the pellet, with mesopore ‘side-roads’ off them to provide active surface area. While optimal fractal-like structures have been proposed from in silico studies (Gheorghiu and Coppens 2004), and some fractal pore spaces synthesised from silica materials (Mayama and Tsujii 2006), for example, these materials have very low hydrothermal stability and rapidly undergo structural collapse in realistic industrial process conditions. Hence, real industrial materials use more robust, but more amorphous, bidisperse structures. A commonly proposed alternative is oxide foam structures with porous walls (Faure et al. 2011). However, for more amorphous structures, the aforementioned issues, with the conventional characterisation and modelling approach to assessing the impact of pore structure on mass transport, arise. Hence, there is a need for the proposed direct experimental methods of assessing the efficiency of the macroporous contribution to mass transport for these materials. In this work a bidisperse foam structure will be used as a case study to illustrate the use of the novel method proposed here for direct experimental evaluation of structure–transport relationships.

## Materials and method

### Material

The industrially produced foam was monolithic in form (as will be seen from the CXT image given below).

### Integrated mercury porosimetry and gas sorption

Approximately 0.2 g of the sample was weighed and placed in a large sample tube made for the gas adsorption rig. The sample tube was then heated to 140 °C while the sample was under vacuum for a minimum of 2 h. The aim of this is to create a standard reference starting point and to remove any physisorbed substances on the pore surface of the sample. The mass of the sample tube and dry sample is weighed and transferred to the Micromeritics ASAP 2010C physisorption rig. The next step was to cool the sample tube to 77 K by manually raising the Dewar, allowing the sample to freeze for approximately thirty minutes and then evacuate the system to remove any physisorbed substances. The rate of adsorption was measured using the constant volume method employing the relevant Micromeritics ASAP 2010C software, as described elsewhere (Hitchcock 2011). Full gas sorption isotherms were also obtained.

Once the initial rate of adsorption and isotherm experiment was finished, mercury was entrapped in the macropore network by performing a standard mercury intrusion/extrusion experiment reaching a desired intrusion pressure for the level of mercury entrapment. For this project a Micromeritics 9500 autopore mercury porosimeter is used. The sample was transferred back to the Micromeritics ASAP 2010C physisorption rig where the rate of adsorption data investigation is repeated. The sample is cooled to 77 K by manually raising the Dewar flask, allowing the sample to freeze for approximately 30 min. This part is important in the post mercury entrapment step as it will freeze the mercury to prevent the mercury vapour from damaging the physisorption apparatus. The rate of adsorption data and isotherm data were then acquired in the same way as before the mercury entrapment.

### Computerised X-ray tomography (CXT)

The foam sample following mercury porosimetry was imaged using a High Resolution X-ray 3D Computed Tomography Microscope Instrument of model VeraXRM-510 (manufactured by Xradia Inc, Pleasanton, CA, USA). The voxel resolution was 8.865 μm.

### NMR cryodiffusometry

For the cryoporometry melting curve experiments the confined water was cooled to 250 K, and the sample was warmed up in steps of 0.3 K per 10 min. The NMR spectrum was taken at each temperature following equilibration. The echo time was chosen such that no signal was obtained when the sample was fully frozen.

For the NMR diffusometry experiment at selected temperatures, a pulsed-field gradient (PFG) sequence was employed with the diffusion time Δ, of 240 ms, and the magnetic gradient pulse length δ, was applied for 90 µs. A range of echo attenuations (R) was obtained by varying the gradient strength *g*. The observed diffusivity was obtained from the negative gradient of a ln*R* vs. *γ* ^*2*^ *g* ^*2*^ *δ* ^*2*^ (∆ − *(δ/3)*) plot. The tortuosity was obtained from the ratio of the bulk diffusivity at the relevant temperature to the observed diffusivity.

### Electron microscopy

The machine used was a FEI XL30 FEG-ESEM.

## Theory

### Mercury porosimetry

In general, the basic data arising from a mercury porosimetry experiment is analysed using the Washburn (1921) Equation. However, it has been shown (Kloubek 1981) that both the contact angle *θ* and surface tension *γ* vary with the radius of curvature of the meniscus, and that the contact angle depends upon whether the meniscus is advancing or receding. More recently (Kloubek 1981; Rigby 2002) expressions for the product *γ.cos θ* have been obtained that incorporate these effects. These expressions have been derived by measuring the pressure *p* at which mercury enters or leaves a model porous medium, with a well-defined structure, for which an independent measure of pore size *r*, such as via electron microscopy, is available (Liabastre and Orr 1978). Insertion of these expressions into the Washburn Equation gives rise to overall relationships of the form:

1 $$r = \frac{{ - A + \sqrt {\left( {A^{2} - 2pB} \right)} }}{p}$$

where *A* and *B* are constants depending on the material, and whether the mercury meniscus is advancing or retreating. The values of *A* and *B* for silica and alumina are given in Table 1. The expressions of the form of Eq. (1) are empirical in origin, and are, therefore, of limited range of applicability (see Table 1), and also contain experimental error (~4 to 5 % (Kloubek 1981).

**Table 1:** Parameters for use in Eq. 1

Material	*A* × 10^3^ (N m^−1^)	*B* × 10^12^ (N)	Range of validity (nm)
Silica (advancing meniscus)	−302.533	−0.739	6–99.75
Silica (retreating meniscus)	−68.366	−235.561	4–68.5
Alumina (advancing meniscus)	−302.533	−0.739	6–99.75
Alumina (retreating meniscus)	−40	−240	4–68.5

### Thermoporometry

Thermoporometry is based upon the phenomenon that the melting and freezing points of fluids are altered when the fluid is confined within a porous solid. In general the shift in the melting point for a small crystal, relative to the bulk, varies inversely with crystal size (Mitchell et al. 2008). For a generic crystal melting within a cylindrical pore the relevant form of the Gibbs–Thompson equation implies the melting point depression ∆*T*_*m*_ is given by:

2 $$\Delta T_{m} = T_{m}^{\infty } - T_{m} \left( x \right) = - \frac{{4\sigma_{sl} T_{m}^{\infty } }}{{x\Delta H_{f} \rho_{s} }}\cos \left( \varphi \right) ,$$

where $T_{m}^{\infty }$ is the bulk melting temperature, *T*_*m*_ *(x)* is the melting point in a pore of diameter *x*, *σ*_*sl*_ is the surface tension, ∆*H*_*f*_ is the bulk enthalpy of fusion, *ρ*_*s*_ is the density of the solid, and *ϕ* is the contact angle (typically assumed to be 180^○^ for liquid–solid). This equation is often simplified to the form:

3 $$\Delta T_{m} = \, k/x,$$

where *k* is the Gibbs–Thompson constant.

## Results

### Electron microscopy

**Fig. 1:**
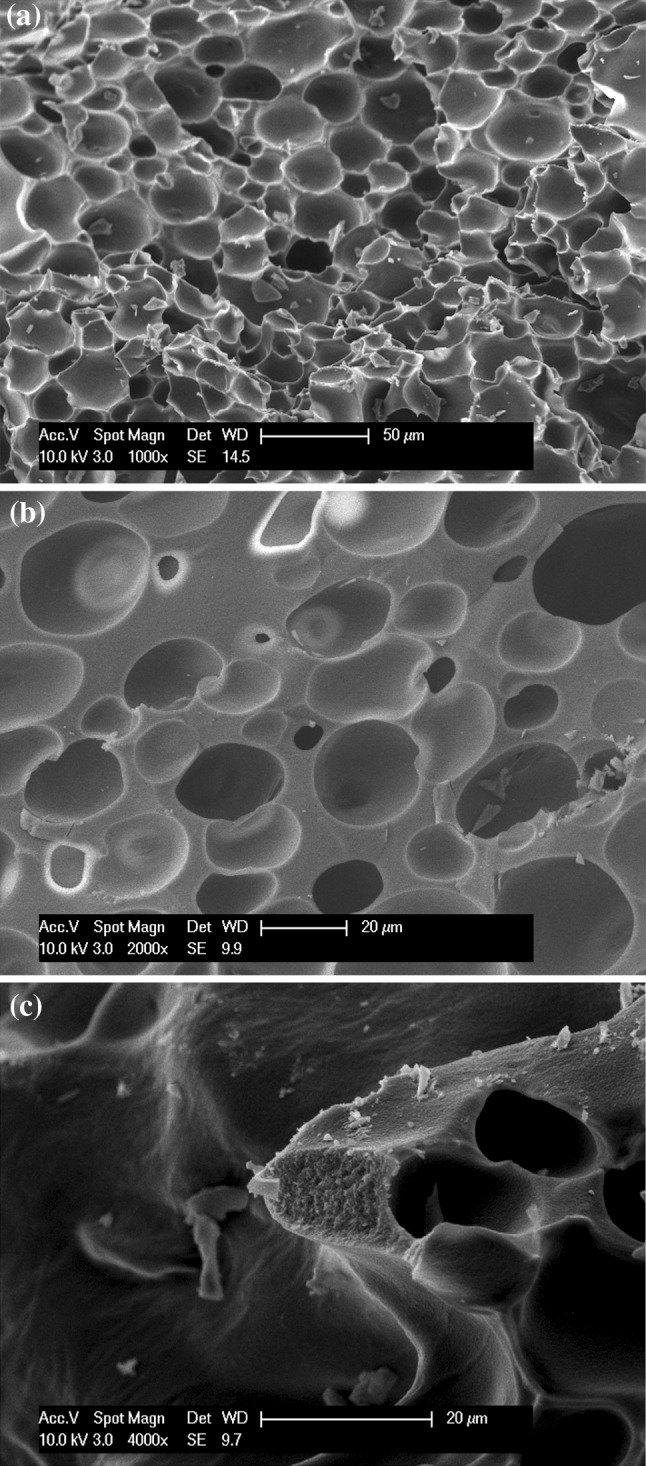
Scanning electron micrographs of different sections **a**, **b** of the foam showing macroporous cells or ‘bubble-shaped’ pores; and **c** a close-up view of foam wall

Figure 1 shows examples of electron micrograph images of a sample of the alumina foam. From Figs. 1a–c) it can be seen that the foam generally consisted of isolated macroporous cells, with few if any indications of inter-cellular windows. There was some spatial variation in the morphology of the sample from place to place, such as thickness of the bubble pore walls, and the electron micrographs in Fig. 1a, b reflect this, since they were taken in different spatial locations. However, in some parts of Fig. 1a a region of thicker pore walls similar to that in Fig. 1b can also be seen. The roughness of the end section of the pore wall in Fig. 1c is suggestive of the presence of intra-wall porosity.

### Integrated gas sorption and mercury porosimetry

**Fig. 2:**
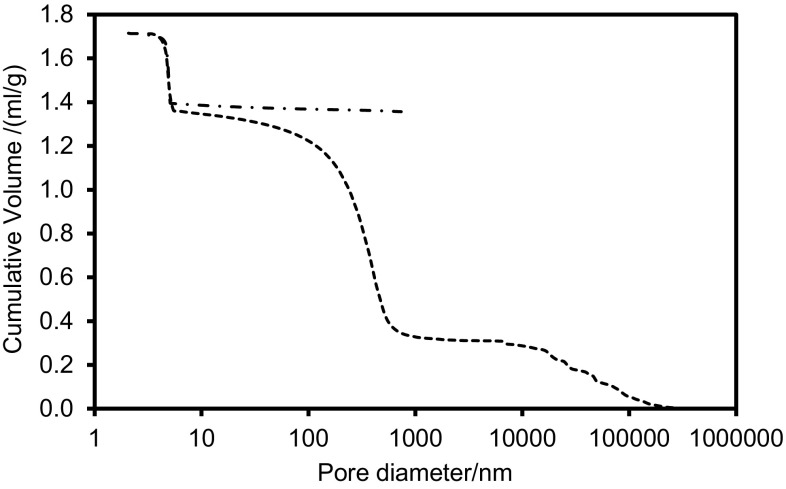
Mercury intrusion (*dashed line*) and extrusion (*dot-dash line*) for a sample of alumina foam analysed using Kloubek correlations (Eq. 1) and parameters for alumina

Figure 2 shows the raw mercury porosimetry data after being analysed with the Kloubek correlations (Eq. 1) using the relevant parameters for alumina. The initial rise in the intrusion curve at very large pore sizes represents intrusion into gaps between sample particles and ruts in the surface. It can be seen that use of the Kloubek correlations leads to the removal of contact angle hysteresis in the raw data, and, thence, a superposition of the intrusion and extrusion curves in the mesoporous region. This suggests that the intrusion into the mesopores is reversible, save for the small amount of entrapment indicated by the deviation of the extrusion curve at the very largest pore sizes present in the mesopore region. Following this deviation the retraction curve is virtually horizontal suggesting that all of the mercury is retained in pores with sizes larger than the point of deviation. The mercury porosimetry data suggests roughly three-quarters of the void volume has been filled with entrapped mercury.

There is less shielding of the macroporosity in the mercury porosimetry data in Fig. 2 compared to what might have been anticipated based on the general lack of connecting macroporous windows for bubble pores in the electron microscopy images. This may be because the bubble pore walls are thin in places and the high mercury pressure may generate larger windows at weak spots to permit early intrusion. These windows would be blocked, however, once the mercury within the bubble is entrapped and frozen. Hence, there would not be any additional openings remaining for the second nitrogen rate of adsorption experiment.

**Fig. 3:**
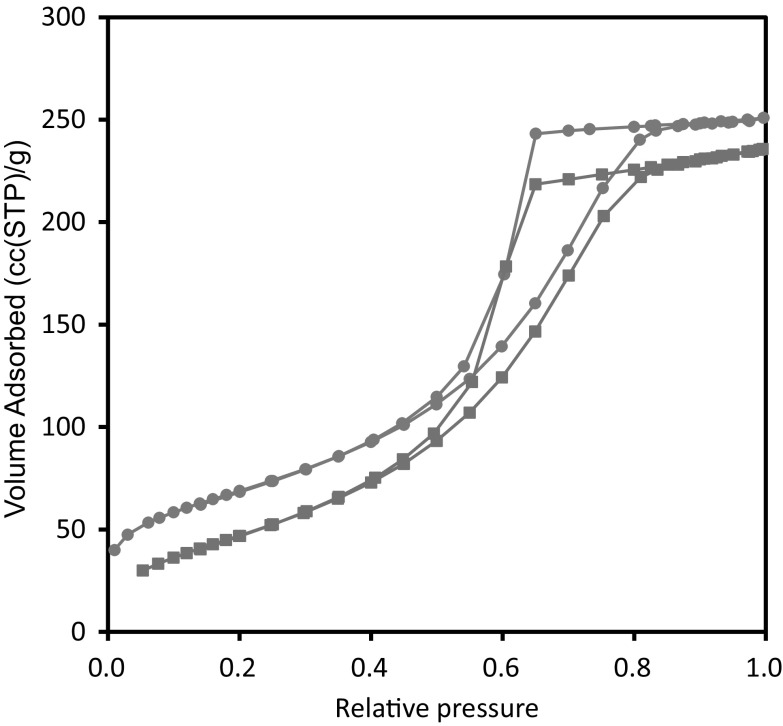
Nitrogen sorption isotherms for foam sample obtained at 77 K before (*filled circle*) and after (*filled square*) mercury entrapment. For the data shown, the amount adsorbed is reported per unit mass of alumina both before and after mercury entrapment to allow these data to be compared on an equivalent basis since the mass of alumina alone remains unchanged following entrapment

Figure 3 shows the nitrogen sorption isotherms obtained for the foam material before and after the mercury porosimetry experiment. From Fig. 3, it can be seen that, following mercury entrapment, there is only a relatively small drop in the volume of nitrogen ultimately adsorbed at the flat plateau at the top of the adsorption isotherm. Since capillary condensation of liquid nitrogen only largely occurs in the mesopores of a material, this finding is consistent with the results from the Kloubek analysis of the mercury porosimetry data that suggested that little or no entrapment occurred in the mesopores. Hence, the gas sorption data also suggests that mercury entrapment is largely confined to the macropores.

### CXT

**Fig. 4:**
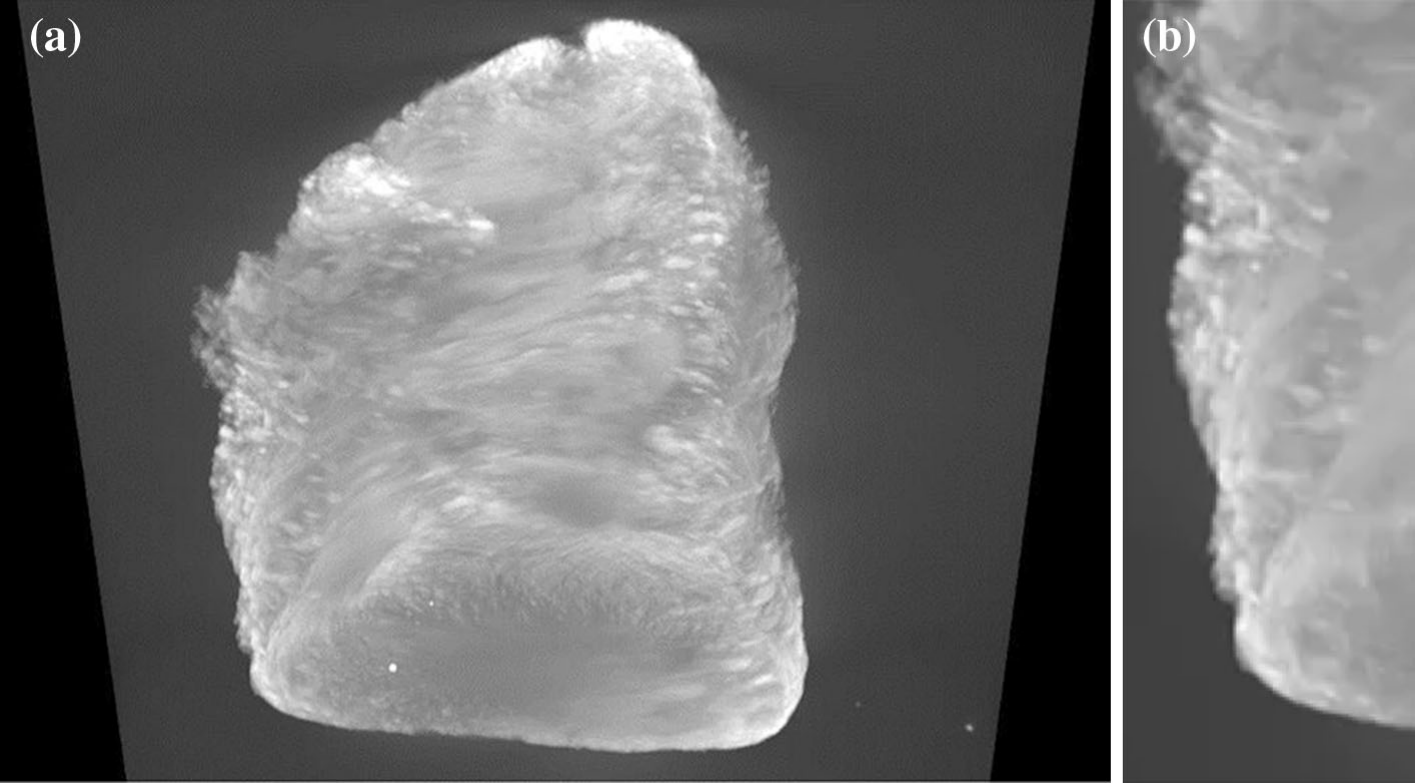
Computerised X-ray Tomography (CXT) image of **a** a monolithic fragment following mercury entrapment, and **b** a close-up view of the left limb of the monolith image. The* bright white* ovoid regions correspond to mercury-filled bubble pores

Figure 4 shows a three-dimensional reconstruction from CXT images of a sample of the foam after discharge from the mercury porosimeter. The brightest white areas of the image correspond to the highest X-ray absorption due to high mercury content. While the centre of the monolith is so thick that little X-ray penetration occurs due to high mercury entrapment, the internal spatial distribution of mercury is more apparent near the limbs of the image where the X-ray path-length is shorter and, thence, absorption less. In the close-up image (Fig. 4b) it can be seen that there are many discrete, white ellipsoidal shapes that are similar in outline to the bubble-like macropores seen in the electron micrographs of the fresh foam given in Fig. 1. It is thus suggested that these white ellipsoidal shapes seen in the CXT image are bubble-like macropores filled with entrapped mercury. Hence, the CXT data is consistent with the suggestion from the mercury porosimetry that the macroporosity retains entrapped mercury.

### Rate of Gas Adsorption

**Fig. 5:**
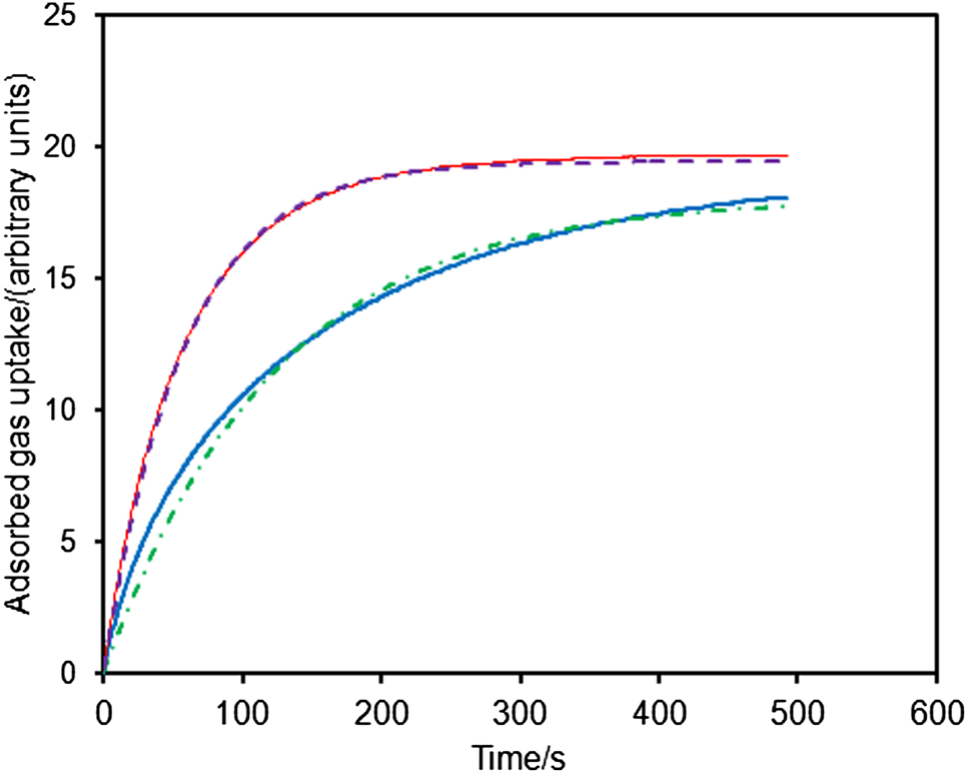
Experimental nitrogen gas uptake against time for foam sample before (*thin solid line*) and after (*thick solid line*) entrapment of mercury, together with fits of the LDF model to these data from before (*dashed line*) and after (*dot-dash line*) entrapment, respectively

Figure 5 shows the experimental gas uptake curves for uptake in the sub-statistical monolayer region (ultimate equilibrium P/P_0_ ≈ 0.068) of the nitrogen adsorption isotherm, such that the pores contained very little adsorbed nitrogen to obstruct access. It was found that the gradients of the isotherms at the equilibrium pressure for the uptake step were such that the corrections to the mass transport coefficient to account for adsorption were within 3 % of each other for before and after mercury entrapment. However, from Fig. 5, it can be seen that the rate of uptake declines much more significantly following mercury entrapment. The experimental uptake curves (for uptakes >50 % of the final plateau) were fitted to the Linear Driving Force (LDF) model (Sircar and Hufton 2000), and, as also shown in Fig. 5, this model was found to give rise to good fits to the data. It was found that the mass transport coefficients (mtcs) were 0.017 s^−1^ before entrapment, and 0.008 s^−1^ after entrapment. Hence, the mtc declined by a factor of ~2 between before and after mercury entrapment.

### NMR cryodiffusometry

**Fig. 6:**
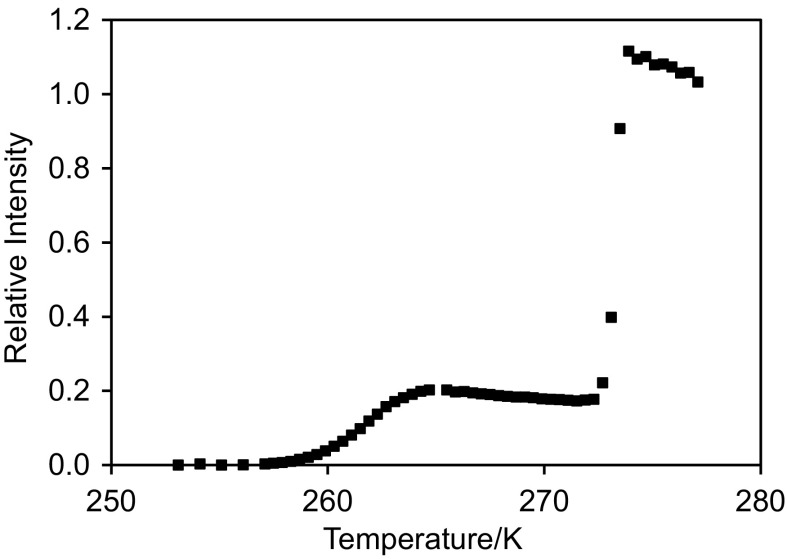
^1^H NMR cryoporometry melting curve for a fully water-saturated sample of alumina foam. The spectral intensity as been renormalized to that of the last point for the fully molten sample

Figure 6 shows the cryoporometry melting curve for a sample of the foam fully saturated with water. It can be seen that there are two main steps in increasing intensity, one at ~259 to 265 K and one at ~272 K. The Gibbs–Thomson parameter is typically taken as 50 K nm^−1^ (based on diameter), for oxide materials (Schreiber et al. 2001). Hence, the mesopore diameters are ~3.6 to 6.3 nm. This is similar to the value obtained from mercury porosimetry shown in Fig. 2. The pore volume fraction of macroporosity from cryoporometry is 0.84, compared to 0.75 from mercury porosimetry.

Table 2 shows the tortuosities obtained for water self-diffusion in the foam when just the water in the mesopores was molten (270 K), and when the whole pore network was molten (274 K). The root-mean square (RMS) (average straight-line) displacement at 274 K was ~35 μm so is larger than typical foam cell size (~20 μm). RMS displacement at 270 K was ~17 μm so was of the same order in size as the wall thickness of foam (in Fig. 1b). The results suggest that molecular diffusion with macroporosity was ~3 times faster than without.

**Table 2:** (PFG) NMR cryodiffusometry results for alumina foam

Temperature (K)	Bulk water diffusivity (m^2^ s^−1^)	Molten pore system diffusivity (m^2^ s^−1^)	Tortuosity
274	1.3 × 10^−9^	7.5 × 10^−10^	1.7
270	9.8 x 10^−10^	1.9 x 10^−10^	5.2

## Discussion

**Fig. 7:**
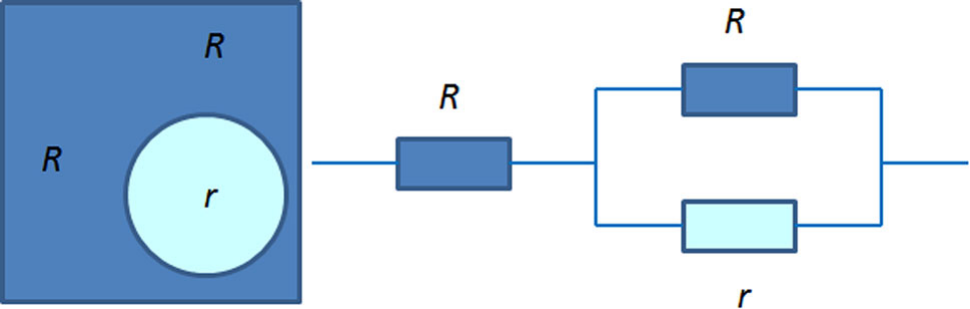
Schematic diagram of simple unit cell model for alumina foam structure and mass transport resistance. The *light shade circle* corresponds to the bubble-like macropore, and the *dark-shaded region* is the mesoporous cell walls

It has been seen that during porosimetry mercury becomes predominantly entrapped in the macroporous bubble-like pores, thereby removing access to them for nitrogen gas. This resulted in a decline in gas uptake rate by roughly a factor of two following porosimetry. However, this factor of the decline in the mass transfer coefficient is less than the factor of decline in the porosity due to mercury entrapment. After mercury entrapment, the gas is largely forced to flow just through the more confined geometry of the mesoporous foam walls, rather than more freely across the macroporous voids. The order of magnitude of the decline in the transport parameter can be rationalised with a simple model. Even though the foam structure is largely random on the macroscopic scale, the key aspects of the structure controlling mass transport can be adequately represented by a simple unit cell model, shown in Fig. 7, which is considered to be periodically repeated in all directions. In this unit cell the pore wall separating one bubble pore from the previous one is represented by a resistance to diffusive flux of size ~*R*. The resistance to the diffusive flux for passage through the foam wall around a bubble-shaped macropore is also of size ~*R*. However, the resistance to the diffusive flux traversing a bubble-shaped macropore is ~*r*. The overall resistance to the passage of the diffusive flux across the structural unit cell (from which the overall foam structure can be built up), is given by:

4 $$R_{tot} = \, R \, + \left[ {\left( {1/R} \right) + \left( {1/r} \right)} \right]^{ - 1} ,$$

where *R*_*tot*_ is the overall resistance of the unit cell. Before mercury entrapment the resistance to the passage of the diffusive flux across one of the bubble macropores would be very much less than that of passage through the mesopore wall around the macropore. Hence, the limiting case then applying would be:

5 $$r \to \, 0,R_{tot} \to \, R.$$

However, once the bubble macropores have been filled with entrapped mercury, the resistance to passage of the diffusive flux that the macropores present would rise dramatically, such that it is very much larger than for the mesoporous walls. In this case the limiting resistance is given by:

6 $$r \, \to \, \infty , \, R_{tot} \to \, 2R$$

It can thus be seen from Eqs. 5 and 6, that, for the resistance network in Fig. 7 representing the foam unit cell, the ratio of network resistance between after entrapment and before is likely to be a factor of ~2. This is in line with the experimental observations reported above. Further, in both limiting cases, the overall resistance is dominated by that of the mesoporous walls. These findings suggests the aforementioned simple picture of the foam structure captures the mass transport properties, and the bubble macropores are likely to be disconnected except via mesopores. The size of the change in the mass transport rates, between when the macropores are available and when they are not, suggests that even though the macroporosity is large it does not control the observed mass transport resistance. The degree of impact of the macropores on mass transport as deduced from the integrated gas sorption experiments is similar in order to that deduced from NMR cryodiffusometry.

The mass transfer coefficient (mtc) scales with the inverse square of particle size so a factor of 2–3 difference in mtc would represent an equivalent particle characteristic dimension (e.g. diameter) change of ~40 to 70 %. A reduction in overall catalyst particle size, for a purely mesoporous material to achieve the same mtc as the foam, would increase bed pressure drop. Further, as demonstrated by natural bone, foam structures are also stronger for the same mass of material, compared to more spatially homogeneous distributions of porosity. However, if the macropore network of the foam was also ‘fully connected’ (percolating) this would be equivalent to having just the parallel section of the resistor network in Fig. 7 (where resistances add reciprocally), so the overall resistance of the pore network would be closer to *r*, i.e. the resistance of the macropores alone. If mass transport were in the Knudsen diffusion regime, that would mean the relative resistance between macro- and meso-pores would scale with pore size (for regions of equivalent porosity and topology), such that the mtc would be increased by a factor of ~100. However, connected macropores may also act like a pervasive fracture network and reduce pellet strength.

## Conclusion

It has been shown that the integrated mercury porosimetry and rate of adsorption technique described here can separately assess the contribution that the macroporosity, that entraps mercury, makes to mass transport, and thereby directly assess the efficacy of that macroporosity in increasing network accessibility. It has been shown that the macropores in the foam material case study improve mass transport by a factor of ~2 to 3, in line with expectations from a simple resistor network model.
